# The complete chloroplast genomes of three Alismataceae species, including the medicinally important *Alisma orientale*

**DOI:** 10.1080/23802359.2024.2320419

**Published:** 2024-03-28

**Authors:** Wen Zheng, Jing Liu, Wenqi Zhao, Zongyi Zhao, Zhiqiong Lan, Jun Wen

**Affiliations:** a School of Pharmacy/College of Modern Chinese Medicine Industry, State Key Laboratory of Southwestern Chinese Medicine Resources, Chengdu University of Traditional Chinese Medicine, Chengdu, China; b College of Life Science, Sichuan Agricultural University, Ya’an, China; c Department of Botany, National Museum of Natural History, MRC166, Smithsonian Institution, Washington, DC, USA

**Keywords:** *Alisma*, Alismataceae, chloroplast genome, herbariomics, phylogenetic analysis

## Abstract

Alismataceae is one of the early diverged families of monocotyledonous plants. We report the complete chloroplast genomes of three *Alisma* species, including *Alisma orientale* (Sam.) Juzep. 1934, *A. subcordatum* Raf. 1908, and *A. triviale* Pursh 1813, of which *A. orientale* is a traditional Chinese medical plant used widely to treat diuretics, diabetes, hepatitis, and inflammation. We sequenced the complete chloroplast genomes with the Illumina Nova-Seq 6000 platform using herbarium collections. The chloroplast genomes of *A. orientale*, *A. subcordatum* and *A. triviale* are 159,861 bp, 160,180 bp, and 159,727 bp in length, respectively. The three chloroplast genomes each contain 113 genes, including four rRNAs, 30 tRNAs genes, and 79 protein-coding genes, and the average GC content is 36.0%. Based on the whole chloroplast genomes of 19 species of Alismataceae and the close allies, the medicinally important *A. orientale* was found to be closely related to another medicinal plant *Alisma plantago-aquatica* L. 1753 in the phylogenetic analysis. The genus *Alisma* was supported to be monophyletic.

## Introduction

Alismataceae is an ancient lineage of monocots whose complex evolutionary history has received much attention in recent years (Li et al. 2022). The genus *Alisma* L. is a cosmopolitan genus of aquatic and wetland plants and contains about 10 species (Björkqvist 1968; Wang et al. 2010). We report the complete chloroplast genomes of three *Alisma* species, *A. orientale* (Sam.) Juzep. 1934 from Eastern Asia, and *A. subcordatum* Raf. 1908, and *A. triviale* Pursh 1813 from North America, using herbarium collections. Of the three species we sampled, *A. orientale* has been valued as important in traditional Chinese medicine, and is native to Eastern and South Asia including China, Korea, Japan, Eastern Russia, India, Myanmar, Nepal, Kashmir, Mongolia, and Vietnam (Han et al. 2013). The tuber of *A. orientale* can be used to treat a variety of diseases, such as diuresis, oliguria, diabetes, hyperlipidemia, hepatitis, and obesity (Liu et al. 2013; Jang et al. 2015; Chinese Pharmacopoeia Commission 2020; Japanese Pharmacopoeia Commission 2021). We hope the genomic information on medicinally important species and their closely related species will lead to better taxonomic delimitations and improve resource conservation and the downstream application of medical resources, which are important for the modern utilization process of traditional natural herbs.

## Materials and methods

Our study used the herbarium collection as source of DNA to perform next-generation sequencing. The sample of *A. orientale* was collected from Bhadwar, Kangra, Punjab, India (*W.N. Koelz 4204*, 30°32′3″N, 74°55′27″E); *A. subcordatum* was collected from Daingerfield Island Marina along the abandoned greenhouse and marshy field, Virginia, U.S.A. (*K.M. Van Neste 412*, 38°49′37″N, −77°02′28″E); and the voucher specimens of the two species are deposited in the US National Herbarium (Dr. Jun Wen, wenj@si.edu); and *A. triviale* was collected from Sandstone Ranch, Douglas County, Colorado, U.S.A. (*J. L. Wingate 13366*, 39°13′29″N, −104°57′7″E), and the voucher specimen is deposited in the KHD Herbarium, Denver Botanic Gardens (Dr. Jennifer Ackerfield, jennifer.ackerfield@botanicgardens.org).

Total genomic DNA was extracted from herbarium specimens using the modified SDS method (Dellaporta et al. 1983; Johnson et al. 2023). An Illumina paired-end DNA library was prepared to use a KAPA HyperPrep Kit (Hoffmann-La Roche, Basel, Switzerland) and then sequenced with the Illumina Nova-Seq 6000 platform (Novogene, Sacramento, CA).

The raw reads were trimmed using Trimmomatic v.0.39 (Bolger et al. 2014), and we used FastQC v0.12.1 (http://www.bioinformatics.babraham.ac.uk/projects/fastqc/) to check the quality of the sequences. For the chloroplast genomes assembly, we employed GetOrganelle v1.7.7 (Jin et al. 2020) based on clean reads without using any reference genomes. All the genes were predicted using CPGAVAS v2.0 (Liu et al. 2012). Subsequently, the chloroplast genomes were annotated and manually checked using Geneious v11.0.18 (Kearse et al. 2012). Chloroplast Genome Viewer (CPGView) software (http://www.1kmpg.cn/cpgview/) (Liu et al. 2023) was used to illustrate the circular genome maps. The annotated genomes have been deposited in GenBank (*A. orientale* accession number: OR773541, *A. subcordatum* accession number: OR773542, *A. triviale* accession number: OR773543).

To investigate the phylogenetic relationships among the three species and their closely related taxa, whole chloroplast genomes of 19 species, including nine species of Alismataceae, nine species of other taxa of Alismatales (Araceae, Hydrocharitaceae, Zosteraceae, Tofieldiaceae, and Butomaceae) as the outgroup specie were downloaded from GenBank. The complete cp genomes were aligned with those of *A. orientale*, *A. subcordatum*, and *A. triviale* using MAFFT v.7.48 (Katoh et al. 2019). We constructed a maximum-likelihood (ML) tree using IQTREE v.1.6 (Nguyen et al. 2015) based on the best fit model TVM + F + G4 and 1000 bootstrap replicates.

## Results

**Figure 1.**
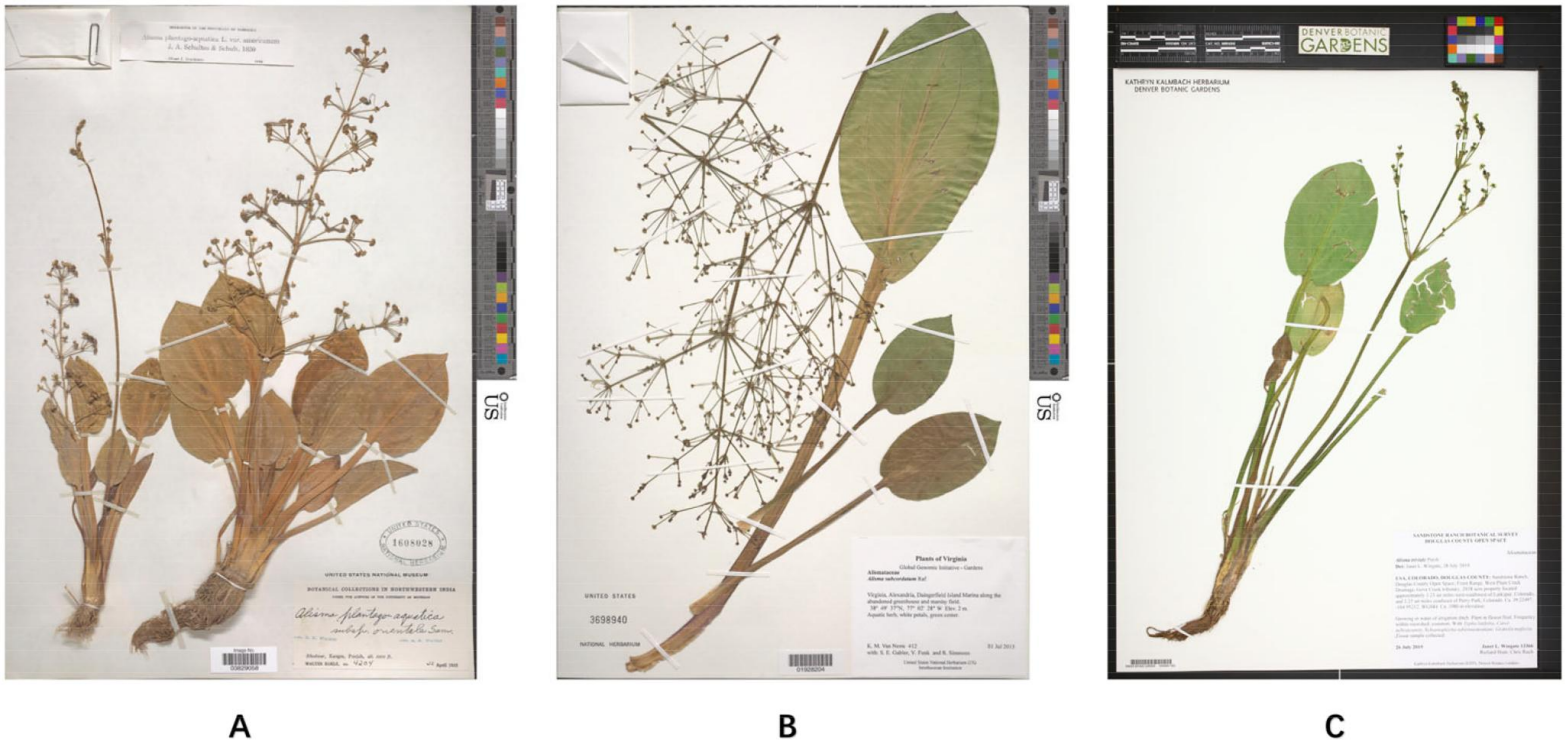
Herbarium specimens used in the *Alisma* chloroplast genome analyses. All three specimens show complete stems, leaves, inflorescences, flowers, and the detailed information of collection and identification. Images of (A) *A. orientale* and (B) *A. subcordatum* are from the official website of the US National Herbarium (https://collections.nmnh.si.edu/search/botany/), and the use of the photos and specimens has been authorized by the Curator, Dr. Jun Wen; and (C) *A. triviale* from the official website of the Kathryn Kalmbach Herbarium of the Denver Botanic Gardens (https://swbiodiversity.org/seinet/collections/harvestparams.php), and the use of the photos and specimens has been authorized by the Curator Dr. Jennifer Ackerfield.

**Figure 2.**
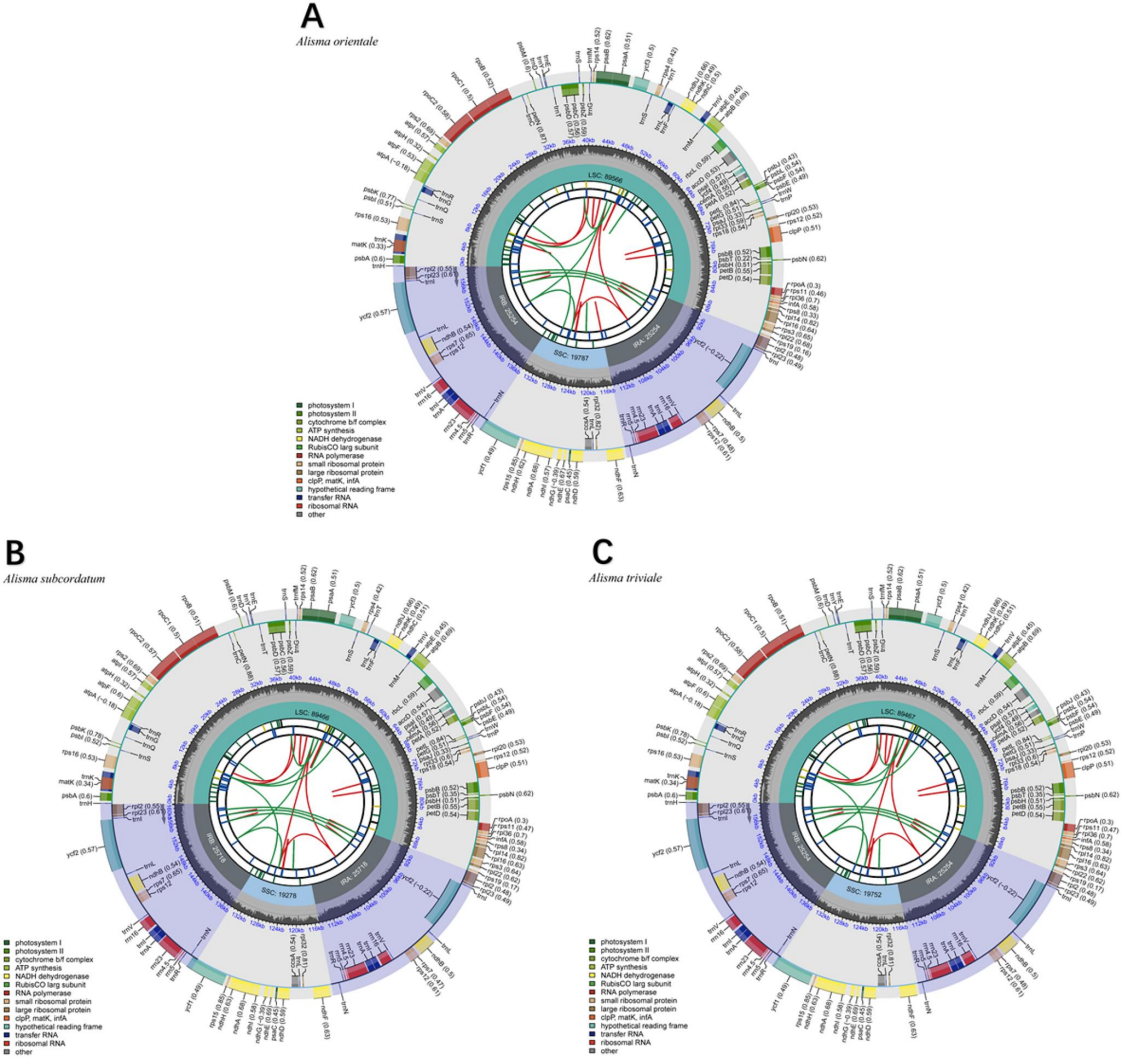
The chloroplast genome map of (A) *A. orientale*, (B) *A. subcordatum*, and (C) *A. triviale*. The map was generated by CPGView. Genes located on the inner and outer of circle are transcribed clockwise and anticlockwise, respectively. The dark grey inner circle indicates GC content. Large single-copy (LSC), small single-copy (SSC), and inverted repeats (IRA and IRB) are indicated in the inner layer. The functional classification of the genes is provided in the bottom left corner.

**Figure 3.**
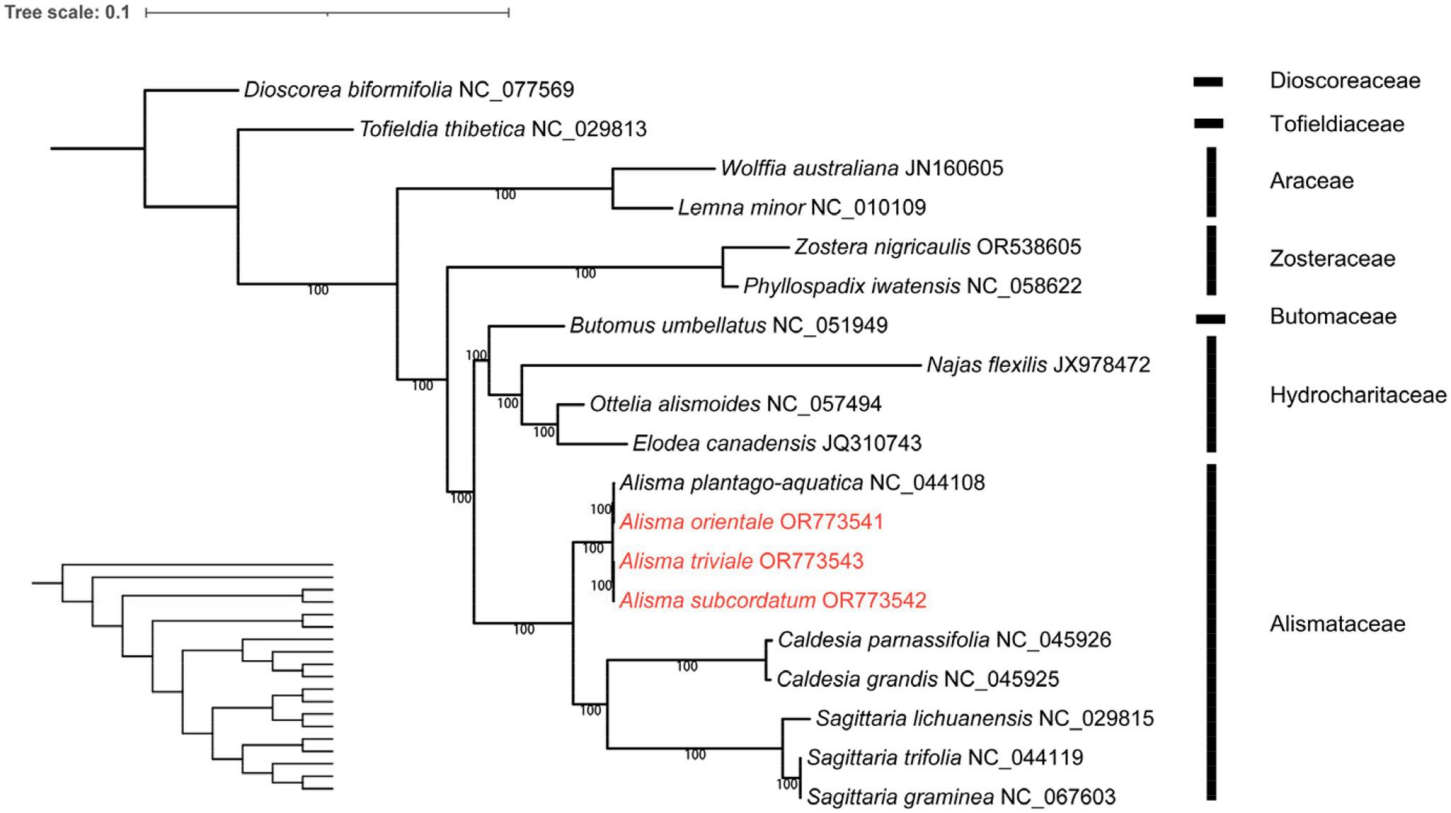
Maximum-likelihood (ML) phylogenetic tree of the order Alismatales based on the complete cp genomes of 18 species in the order, with *Dioscorea biformifolia* (Dioscoreaceae) as the outgroup. The scale bar represents the number of substitutions per site. The bootstrap method used was SH-aLRT. The number above the line represents ML bootstrap value. The specie names colored in red represent the three newly sequenced chloroplast genomes (*Alisma orientale* OR773541, *A. subcordatum* OR773542 and *A. triviale* OR773543). The following sequences were used: *Dioscorea biformifolia* NC_077569 (unpublished), *Tofieldia thibetica* NC_029813 and *Sagittaria lichuanensis* NC_029815 (Luo et al. 2016), *Wolffia australiana* JN160605 (Wang and Messing 2011), *Lemna minor* NC_010109 (Mardanov et al. 2008), *Zostera nigricaulis* OR538605 (unpublished), *Phyllospadix iwatensis* NC_058622 (Chen et al. 2021), *Butomus umbellatus* NC_051949 (Yang and Liu 2019), *Najas flexilis* JX978472 (Peredo et al. 2013), *Ottelia alismoides* NC_057494 (Guo et al. 2020), *Elodea canadensis* JQ310743 (Huotari and Korpelainen 2012), *Alisma plantago-aquatica* NC_044108 and *Sagittaria trifolia* NC_044119 (Liang et al. 2019), *Sagittaria graminea* NC_067603 (unpublished), and *Caldesia parnassifolia* NC_045926 and *C. grandis* NC_045925 (Mwanzia et al. 2019).

We viewed the assembly of the chloroplast genome by mapping reads to the assembled chloroplast genome sequence using Geneious and measuring the depth of coverage (Supplementary Figure S1a–S1c). The complete genome circular assembly map was checked by Bandage v. 0.8.1 (Supplementary Figure S2a–S2c). The chloroplast genome of *Alisma orientale* was 159,861 bp in length, the genome of *A. subcordatum* was 160,180 bp in length and that of *A. triviale* was 159,727 bp in length (Figure 1). The sequences had a typical quadripartite structure containing a large single-copy (LSC) region, a small single-copy (SSC) region, and a pair of inverted repeat (IR) regions (Figure 2). The chloroplast genomes sequence of the three species each had 113 unique genes, including four rRNAs, 30 tRNAs genes, and 79 protein-coding genes. Among these genes, 13 cis-splicing genes were discovered. The trans-splicing gene *rps12* had three unique exons (Supplementary material, Figure S3a–S3c). The overall GC content of the whole genome was 36.0%. The phylogenetic analysis showed that *A. orientale* clustered with *Alisma plantago-aquatica* L. 1753, *A. triviale* clustered with *A. subcordatum*, and *Alisma* was sister to the clade of *Sagittaria* and *Caldesia*. Alismataceae is sister to the Hydrocharitaceae – Butomaceae clade of Alismatales (Figure 3).

## Discussion and conclusions

The whole chloroplast genomes of the three *Alisma* species generated in this study were consistent with the published genome structure of *A. plantago-aquatica* (NC_004108) (Liang et al. 2019), and the GC content was slightly different from that of *A. plantago-aquatica*, separated by 0.4%. These four species of *Alisma* formed a monophyletic group, with *A. plantago-aquatica* sister to the medicinally important *A. orientale*, and the two North America species *A. subcordatum* and *A. triviale* were sister to each other. This view was supported by the phylogenetic analysis based on the nuclear region. The close phylogenetic relationship between the medicinally important *A. orientale* and the widespread *A. plantago-aquatica* was in agreement with the Bayesian inference tree of *Alisma* based on chloroplast DNA loci (*matK*, *ndhF*, *psbA-trnH*, and *rbcL*) (Ito and Tanaka 2023). The sister relationship between Alismataceae and the clade of Butomaceae and Hydrocharitaceae was supported by the nuclear phylogeny (Timilsena et al. 2022). The core Alismatales consisting of Alismataceae, Hydrocharitaceae, Butomaceae, Zosteraceae, and Araceae was sister to Tofieldiaceae of the order. The higher-level phylogenetic results were largely consistent with the genus level phylogeny that sampled 10 species of Alismatales based on 79 protein-coding genes (Luo et al. 2016; Liang et al. 2019). Further studies are required to resolve the lower infrageneric relationships of the small medicinally important genus *Alisma*. We anticipate that our findings here will stimulate further studies on *Alisma* and its close relatives and contribute to the resource conservation, molecular evaluation, and identification, and the exploration and utilization of natural pharmacodynamic components of *Alisma* species.

Our study also shows the importance of herbarium collections in the new age of genomics and big data. Herbarium specimens are an important source for biodiversity science and can be effectively used for extracting chloroplast genome and other genomic data (herbariomics) to address questions on species delimitation and utilization of plant medicinal resources (Jiang et al. 2022; Duan et al. 2023; Wen et al. 2023).

## Supplementary Material

Supplemental Material

## Data Availability

The authors have deposited all the raw data in GenBank, which have all been activated. The data that support the findings of this study are openly available in GenBank at https://www.ncbi.nlm.nih.gov/ under the accession number OR773541, OR773542, and OR773543. The associated BioProject, SRA, and BioSample numbers are PRJNA1037010, SRX22416385 (*Alisma orientale*), SRX22416386 (*Alisma subcordatum*), SRX22416387 (*Alisma triviale*) and SAMN38155281 (*Alisma orientale)*, SAMN38155282 (*Alisma subcordatum*), SAMN38155283 (*Alisma triviale*), respectively.
