# Supplementary material for: The complete chloroplast genomes of three Alismataceae species, including the medicinally important Alisma orientale
Source: Mitochondrial DNA B Resour. 2024 Mar 28;9(3):385–9. doi: 10.1080/23802359.2024.2320419 (PMC10984228; doi:10.1080/23802359.2024.2320419)
Supplement: Supplemental Material [file TMDN_A_2320419_SM1054.docx]

**Supplementary Materials**


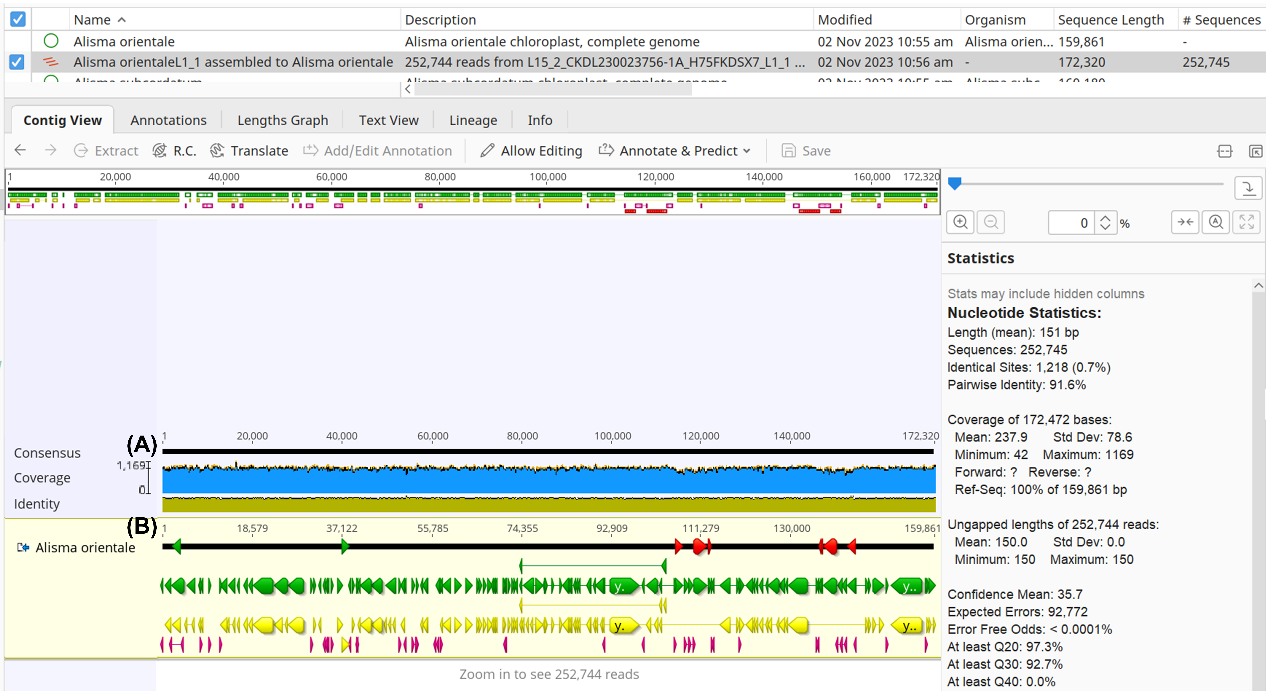


**Fig. S1a:** Mapping of the quality of the chloroplast genome assembly of *Alisma* *orientale* (OR773541). Depth of coverage plot (A) and physical map of cpDNA (B).


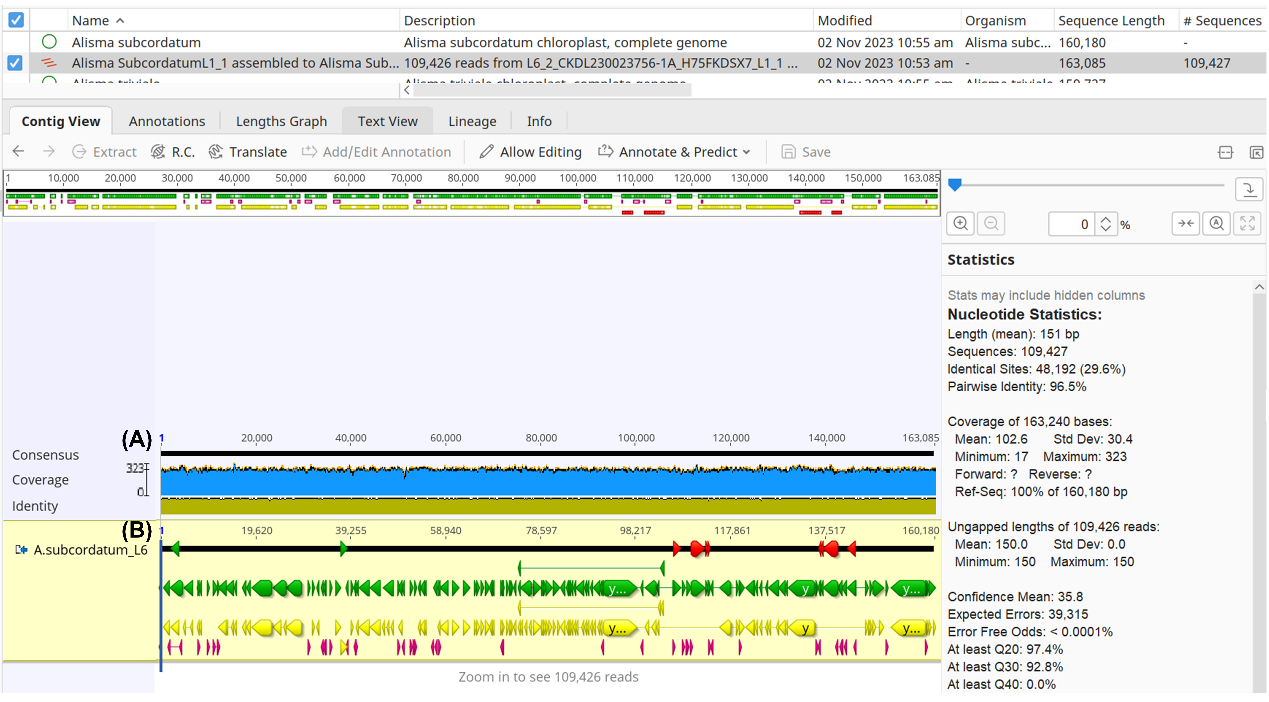


**Fig. S1b:** Mapping of the quality of the chloroplast genome assembly of *Alisma subcordatum* (OR773542). Depth of coverage plot (A) and physical map of cpDNA (B).


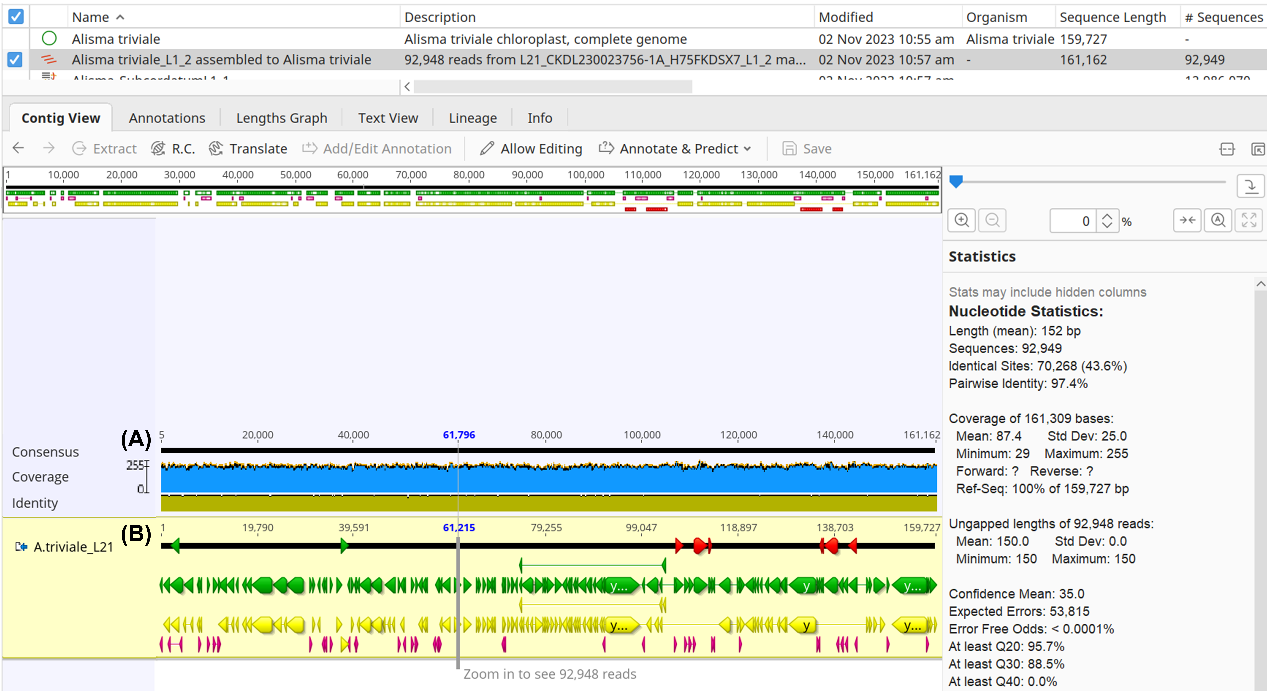


**Fig. S1c:** Mapping of the quality of the chloroplast genome assembly of *Alisma triviale* (OR773543). Depth of coverage plot (A) and physical map of cpDNA (B).

**Note:** In order to understand the sequencing and assembly quality, we counted the coverage of each locus using Geneious v11.0.18 and the final results are shown above.


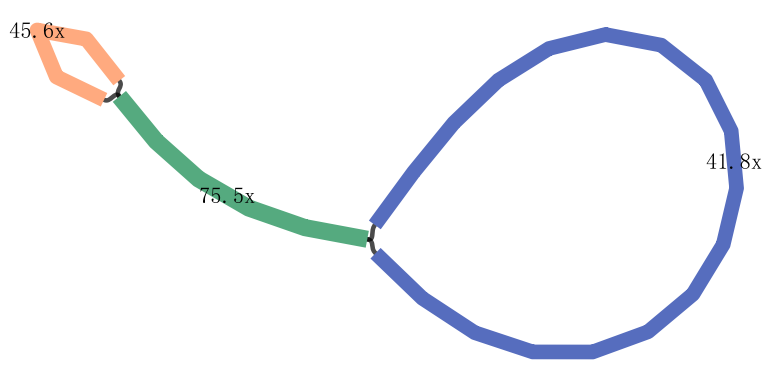


**Fig. S2a:** Circular mapping of the chloroplast genome of *Alisma orientale* (OR773541).


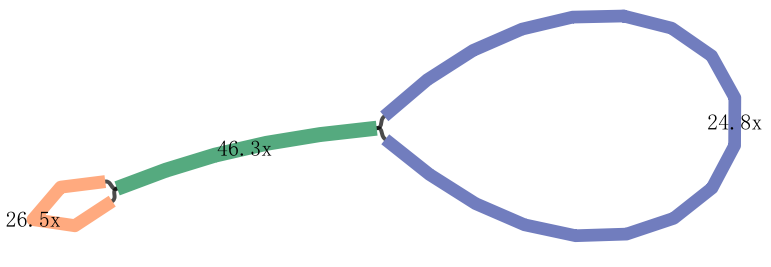


**Fig. S2b:** Circular mapping of the chloroplast genome of *Alisma subcordatum* (OR773542).


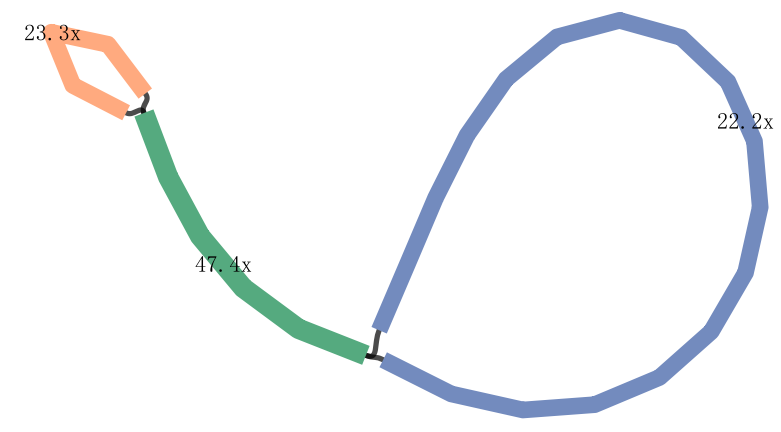


**Fig. S2c:** Circular mapping of the chloroplast genome of *Alisma triviale* (OR773543).

**Note:** Complete circular assembly graph was checked by Bandage v. 0.8.1. The large blue ring represents the large single-copy region, the small orange ring represents the small single-copy region, and the thick green line represents the inverted repeat region. The three sections are tightly connected and represent the chloroplast genome assembled into a circle.


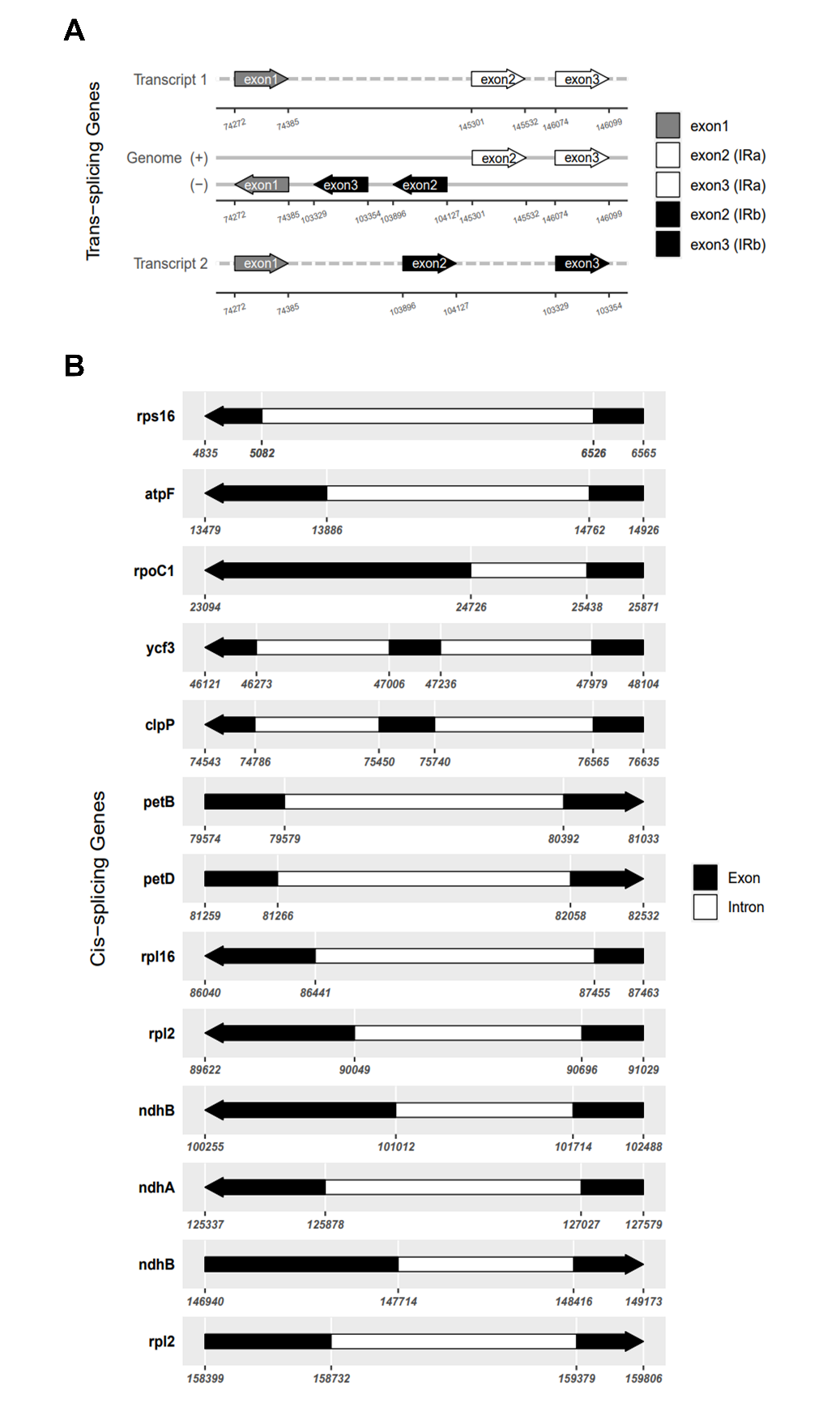


**Fig. S3a:** Physical map of the (A) trans-splicing gene *rps12* and (B) cis-splicing genes in the chloroplast genome of *Alisma orientale* (OR773541).


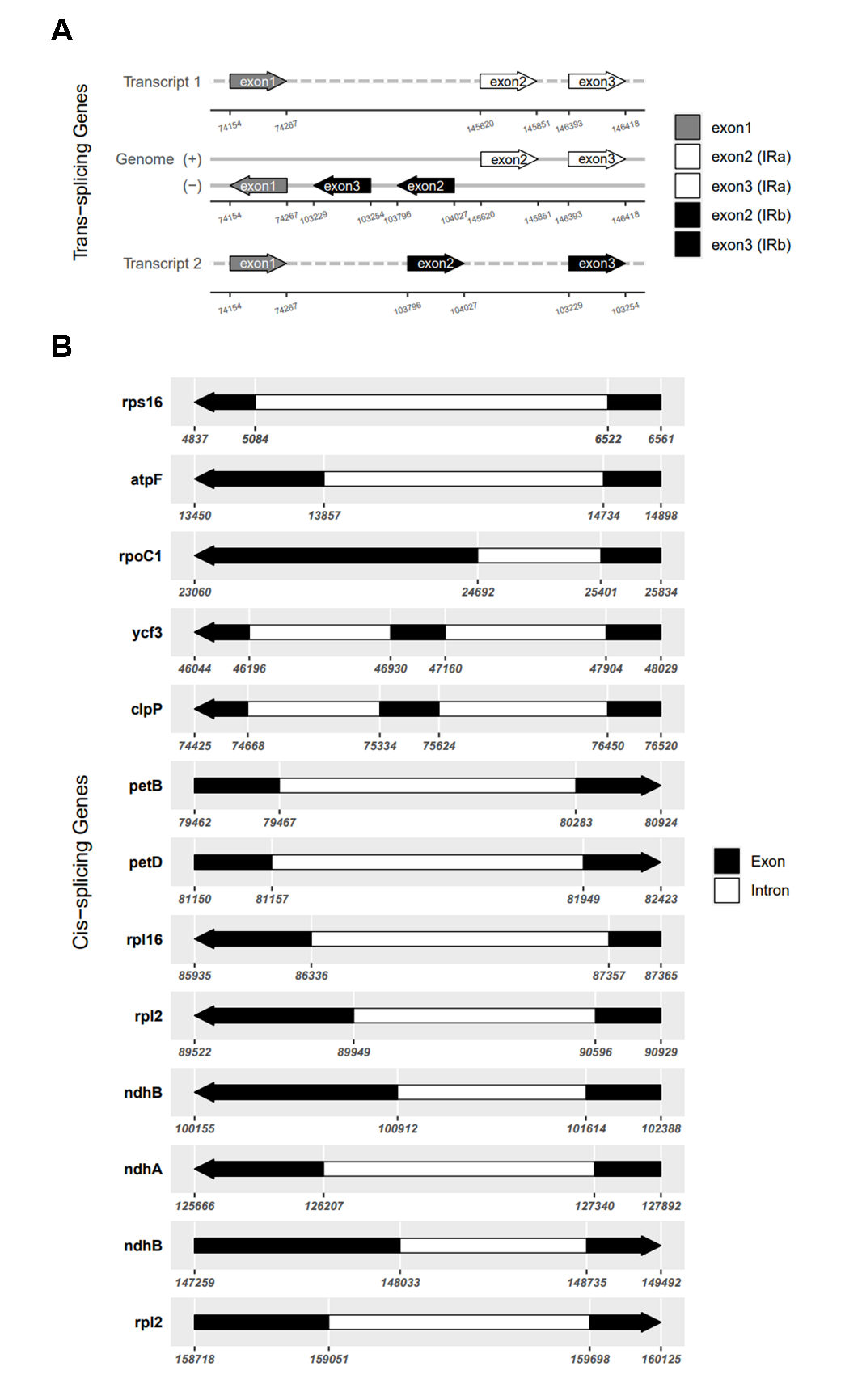


**Fig. S3b:** Physical map of the (A) trans-splicing gene *rps12* and (B) cis-splicing genes in the chloroplast genome of *Alisma subcordatum* (OR773542).


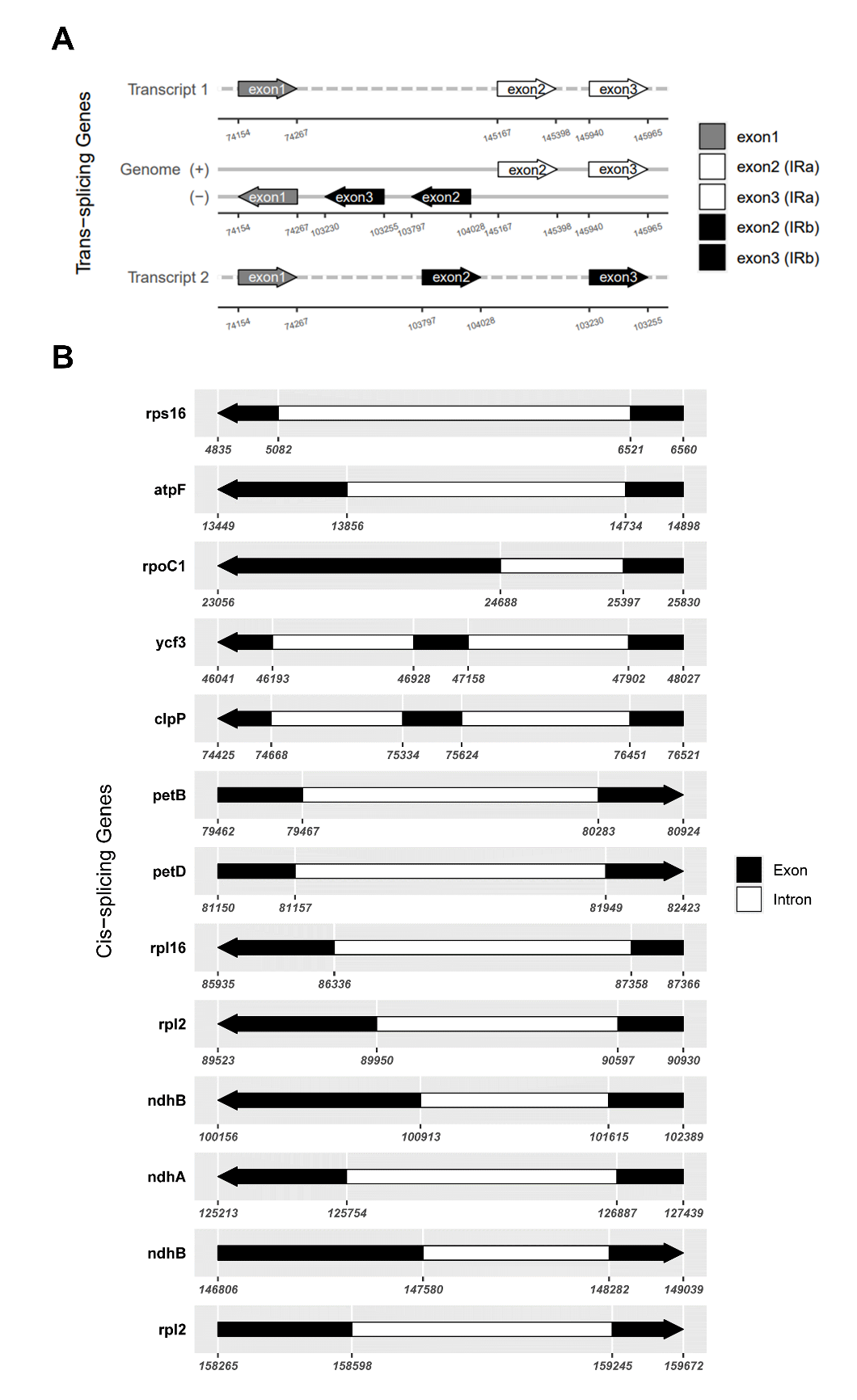


**Fig. S3c:** Physical map of the (A) trans-splicing gene *rps12* and (B) cis-splicing genes in the chloroplast genome of *Alisma triviale* (OR773543).

**Note:** Exons and introns are shown in black and white, respectively. The direction of the genes is represented by arrows. The physical map was constructed using CPGview.
